# Is Extraordinary Response and Long-Term Remission of Metastatic Castration-Resistant Prostate Cancer (mCRPC) After [¹⁷⁷Lu]Lu-PSMA Radioligand Therapy Due to an Immunomodulatory Effect (Radiovaccination)? A Dual Center Experience on Super-Responders

**DOI:** 10.3390/cancers17030476

**Published:** 2025-01-31

**Authors:** Masha Maharaj, Elisabetta Perrone, Ralph M. Wirtz, Lucille Heslop, Trisha Govender, Nisaar A. Korowlay, Kriti Ghai, Tanay Parkar, Richard P. Baum

**Affiliations:** 1 Umhlanga Molecular Imaging & Therapy Centre of Excellence, Department of Nuclear Medicine, Netcare Umhlanga & Hibiscus Hospitals, Durban 4320, South Africa; trisha.govender.tg@gmail.com; 2 Department of Nuclear Medicine, Loveworld Medical Centre, Lagos 100271, Nigeria; 3 Institute of Nuclear Medicine, Università Cattolica del Sacro Cuore, 00168 Rome, Italy; bets.perrone@gmail.com; 4 CURANOSTICUM Wiesbaden-Frankfurt, Center for Advanced Radiomolecular Precision Oncology, 65191 Wiesbaden, Germany; ghai@curanosticum.de (K.G.); parkar@curanosticum.de (T.P.); 5 Stratifyer Molecular Pathology GmbH, 50935 Cologne, Germany; ralph.wirtz@stratifyer.de; 6 The Oncology Centre, Peter Mokaba Ridge, Overport, Durban 4001, South Africa; hesloplucille37@gmail.com; 7 Division of Nuclear Medicine, Tygerberg Hospital, Stellenbosch University, Stellenbosch 7500, South Africa; nak@sun.ac.za; 8 International Centers for Precision Oncology (ICPO), 88214 Ravensburg, Germany

**Keywords:** PSMA, PSMA radioligand therapy (PRLT), radiovaccination, antitumor immunity, abscopal effect, immune induction, immunomodulatory

## Abstract

Background: Prostate-specific membrane antigen (PSMA)-directed radioligand therapy (PRLT) with Lutetium-177 ([^177^Lu]Lu-PSMA) is a safe and effective treatment for metastatic castration-resistant prostate cancer (mCRPC). The aim of our study was to evaluate clinical variables of patients with extreme response to PRLT and to assess its immunomodulatory potential. Methods: This retrospective study included 36 patients from two centers achieving extreme response after [¹⁷⁷Lu]Lu-PSMA PRLT. The primary outcomes were the duration of maintained response in months (MR) and improvement post-therapy—clinically, serologically, and on molecular (PET/CT) imaging. We examined several variables, including pathology, gene sequencing, baseline PSA, Gleason score, prior therapies, number of PRLT cycles, and pattern of disease, to identify potential factors that may influence the extreme response. Results: Between 2018 and mid-September 2024, 36 men with mCRPC received a mean of three cycles of [^177^Lu]Lu-PSMA PRLT. Patients were subgrouped according to clinical variables versus MR. A total of 17 patients had ≥12 months MR (17/36, 47%). The longest duration of MR was 99 months and a mean of 17.44 months (95% CI 10.05–24.84). Previous lines of treatment were evaluated for MR, *p* = 0.172. Pattern of disease (bone, lymph node, liver, and peritoneal) was evaluated for MR, *p* = 0.721. The Gleason score was evaluated for MR, *p* = 0.871. Patients with known BRCA sequencing status (n = 12) were analyzed with mean MR: BRCA1/2 wild-type, 6/12 (50%), 6.67 months; BRCA 1/2 negative, 1/12 (8.33%), 7 months; BRCA germline negative and somatic positive, 1/12 (8.33%), 36 months; BRCA germline negative, somatic negative, 2/12 (16.67%), 27 months; and BRCA 2 positive, 2/12 (16.67%), 43 months. Conclusions: We propose there may be intrinsic mechanisms suggesting the immunomodulatory enhancement of ionizing radiation, primarily driving extreme responses.

## Simple Summary

Ionizing radiation has been shown to enhance antitumor immunity, a concept first described in 1953 as the “abscopal effect” in external beam radiation therapy (EBRT). However, EBRT targets only specific tumor lesions. Radioligand therapy, on the other hand, involves a radionuclide linked to a targeting molecule (e.g., monoclonal antibodies or small molecules) that can target all tumor lesions, regardless of their location, with minimal damage to healthy tissue. This method can also induce immunomodulatory effects, such as increasing the presentation of neoantigens and tumor-associated antigens, leading to an in situ “vaccination” effect. In our study of 36 patients with metastatic castration-resistant prostate cancer (mCRPC) treated with [^177^Lu]Lu-PSMA radioligand therapy (PRLT), persistent therapeutic responses were observed, including clinical improvement, biochemical response (PSA levels), and imaging responses. These findings suggest that radioligand therapy’s immunomodulatory effects contribute to its enhanced therapeutic potential beyond the effects of radiation alone.

## 1. Introduction

Metastatic castration-resistant prostate cancer (mCRPC) represents the most aggressive and fatal stage of prostate cancer progression. Treatment strategies that have demonstrated improvements in survival for mCRPC patients include chemotherapy agents such as docetaxel and cabazitaxel, androgen-axis pathway inhibitors like abiraterone and enzalutamide, and radionuclide therapy for managing skeletal metastasis with Radium-223 dichloride. Prostate-specific membrane antigen (PSMA) is significantly overexpressed in mCRPC, making it an ideal target for both imaging and therapeutic interventions in the management of this condition. PSMA-directed radioligand therapy (PRLT) with Lutetium-177 ([^177^Lu]Lu-PSMA) offers a promising treatment for metastatic castration-resistant prostate cancer (mCRPC) patients who have not responded to hormone therapy or chemotherapy. Retrospective data indicate that PRLT is generally safe and leads to favorable clinical outcomes [1,2,3,4]. The VISION trial [5] demonstrated that [^177^Lu]Lu-PSMA-617, combined with standard care, significantly prolonged both progression-free survival (PFS; median, 8.7 vs. 3.4 months) and overall survival (OS; median, 15.3 vs. 11.3 months) in mCRPC patients compared with supportive care alone, outperforming standard treatments like chemotherapy with docetaxel and cabazitaxel (5.1 and 3.9 months, respectively) [6,7].

There are reports of remarkable response to [^177^Lu]Lu-PSMA therapy [1,4] in mCRPC. Ionizing radiation has been shown to enhance antitumor immunity, a concept first described in 1953 by Dr. Mole as the “abscopal effect” in external beam radiation therapy (EBRT) [8]. Radioligand therapy (RLT) differs from EBRT in that it targets all tumor lesions, regardless of location, with minimal harm to healthy tissue. Ionizing radiation in RLT can also enhance the immune response. It promotes the presentation of neoantigens and tumor-associated antigens, triggering an in situ “vaccination” effect [9]. We assessed patients from two theragnostic centers, representing a diverse range of ethnicities and nationalities. The aim of our study was to evaluate multiple clinical variables of patients with observed enhanced response to [^177^Lu]Lu-PSMA therapy to assess the immunomodulatory potential of radioligand therapy as a form of “vaccination”.

## 2. Materials and Methods

This study is a retrospective analysis of dual-center experiences in treating mCRPC patients with [^177^Lu]Lu-PSMA PRLT at CURANOSTICUM Wiesbaden-Frankfurt, Center for Advanced Radiomolecular Precision Oncology, DKD Helios Klinik (Wiesbaden, Germany) and at the Umhlanga Molecular Imaging & Therapy Centre of Excellence, Department of Nuclear Medicine at Netcare Umhlanga & Hibiscus Hospitals (KwaZulu Natal, South Africa). We pooled data from 2018 to mid-September 2024, including consecutive patients of any age and Eastern Cooperative Oncology Group performance status, all with histopathological confirmed adenocarcinoma of the prostate, treated with one or more cycles of [^177^Lu]Lu-PSMA PRLT (dose range 5.5–7.4 GBq) administered intravenously. The number of treatment cycles was never predetermined but decided according to post-therapy imaging, clinical and biochemical response, and the patients’ well-being. Data about safety, including hematological, renal, and hepatic toxicity, although collected, were not systematically reported and included in this study analysis. Previous treatments included taxane-based chemotherapy, androgen receptor-axis inhibitors, and Radium-223 dichloride. The primary outcomes were the duration of maintained response (MR) and improvement post-therapy—clinically, serologically (PSA decrease or normalization, known as biochemical response), and on molecular and morphological imaging—to evaluate the prolonged therapeutic effect. We defined “Super-Responders” both patients who achieved complete remission of the disease after treatment and patients who demonstrated an extreme response to treatment at a specific site of metastatic disease (e.g., liver tumor involvement), as observed at post-therapeutic SPET/CT or follow-up [^68^Ga]Ga-PSMA or [^18^F]F-PSMA PET/CT, according to molecular (both via SPET/CT and PET/CT) imaging criteria of response (THERCIST) [10].

## 3. Results

**Figure 1:**
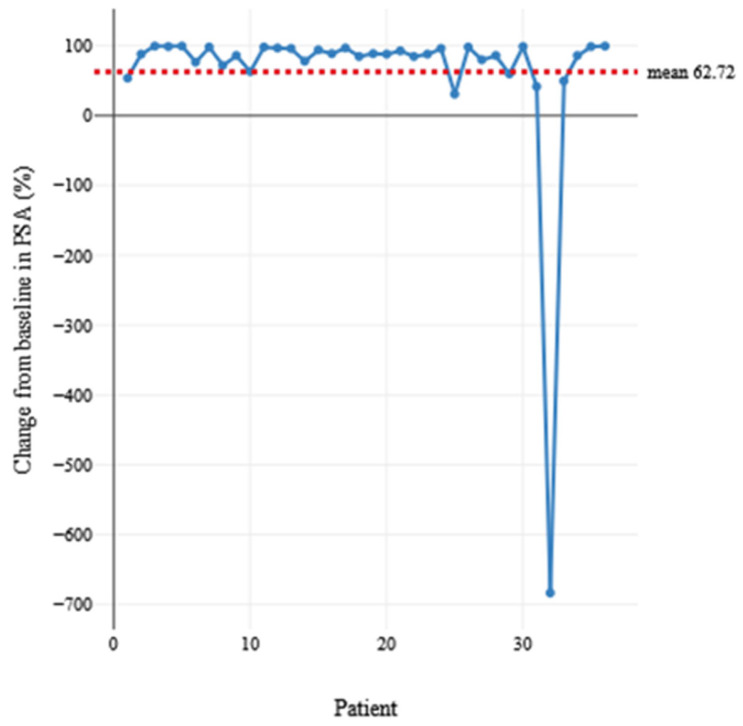
I-Chart illustrating individual %PSA decline post-[^177^Lu]Lu-PSMA PRLT response with mean 67.72% (red).

Between 2018 and mid-September 2024, 36 men [Table S1] with mCRPC received a mean of three cycles of [^177^Lu]Lu-PSMA PRLT. The mean age of the patients at the time of the first cycle was 71.78 years, *p* = 0.224 [Table 1], and baseline PSA values ranged from 0.04 ng/mL to 1345 ng/mL. The mean %PSA decline was 62.72 (95% CI 19.03–106.41), *p* = 0.004. A total of 33 of 36 patients (92%) had a ≥50% PSA decline [Figure 1]. The baseline PSA (ng/mL) was compared to PSA (ng/mL) using the paired *t*-test method. This was found significant, *p* = 0.002 [Table 2]. Patients were subgrouped according to clinical variables versus number of months of MR: ≤6 months (11/36, 31%); >6 months to 12 months (10/36, 28%); >12 months (15/36, 42%) [Table 3]. The longest duration of MR after [^177^Lu]Lu-PSMA PRLT was 99 months and a mean of 17.44 (95% CI 10.05–24.84), *p* ≤ 0.001. The highest number of cycles to complete response (five and six cycles) was observed in three patients. The total number of cycles was assessed against MR, *p* = 0.813 [Table 4]. Previous lines of treatment were evaluated against MR (months) (*p* = 0.172). Prior therapies included docetaxel in 7 patients (19.4%), cabazitaxel in 5 patients (13.9%), therapy with abiraterone in 10 patients (28%), enzalutamide in 11 patients (31%), and EBRT in 15 patients (41.7%) [Table 5]. Pattern of disease (bone, lymph node, hepatic, and peritoneal) was evaluated for MR in months and mean %PSA decline, *p* = 0.721 [Table 5]. Bone metastases were identified in 27/36 (75%), with a mean-maintained response (MMR) of 15.1 months and mean %PSA decline of 85.05%. Lymph node metastases (LNM) were identified in 27/36 (75%), with a MMR of 17.5 months and mean %PSA decline of 84.48% (excluding one patient with increased %PSA level and nodal metastases). Hepatic metastases were identified in 6/36 (16.67%), with an MMR of 4.33 months and mean %PSA decline of 66.0%. Peritoneal metastases were identified in 2/36 (5.6%), with an MMR of 4.5 months and mean %PSA decline of 69.50%. The Gleason score was evaluated against MR, *p* = 0.871 [Table 6]. Patients with known BRCA sequencing status (n = 12) were analyzed. BRCA1/2 wild-type, 6/12 (50%), MMR 6.67 months; BRCA 1/2 negative, 1/12 (8.33%), MMR 7 months; BRCA germline negative and somatic positive, 1/12 (8.33%), MMR 36 months; BRCA germline negative and somatic negative, 2/12 (16.67%), MMR 27 months; BRCA 2 positive, 2/12 (16.67%), MMR 43 months [Table 7]. A total of 17 patients had ≥12 months MR (17/36, 47%). Table 8 tabulates the clinical variables for these patients with prior therapies, Gleason score, pathology and sequencing, pattern of disease, number of cycles to response, MR (months), and individual %PSA decline.

## 4. Discussion

In our dual-center retrospective analysis, we report a series of 36 mCRPC patients treated with [¹⁷⁷Lu]Lu-PSMA radioligand therapy, achieving extreme response after treatment. The mean-maintained response (MMR, months) was 17.44 months (95% CI 10.05–24.84). The mean PSA decline was 62.72% [Figure 1]. A total of 33 of 36 patients (92%) had a ≥50% PSA decline. The baseline PSA (ng/mL) was compared to PSA (ng/mL) using the paired *t*-test method. This was found significant, *p* = 0.002. One patient with recorded aberrant increased PSA (0.41 ng/mL) at time of response compared to baseline PSA (0.06 ng/mL) was a 59-year-old male with mCRPC Gleason score 8 (4 + 4) and LNM, treated with prior therapy of prostatectomy, salvage EBRT of prostate bed and pelvic lymph pathway, therapy with Leuprorelin, Apalutamid, and Darolutamid. This patient had 19 months of maintained complete response [Table 8]. The elevated PSA at time of response was attributed to tumor flare. We examined several variables, including pathology, sequencing, baseline PSA level, Gleason score, prior therapies, number of cycles, and pattern of disease, to identify potential factors that may have influenced on the extreme response to therapy [Table 1, Table 2, Table 3, Table 4, Table 5, Table 6, Table 7 and Table 8]. An extreme response to [^177^Lu]Lu-PSMA PRLT was found to be independent of variability in uptake distribution, intensity of radiotherapeutic uptake, baseline PSA levels, age, previous treatments, race/nationality, Gleason score, BRCA expression, disease pattern, injected dose of [^177^Lu]Lu-PSMA, and number of therapy cycles. In the analysis, we observed that three individual clinical variables were associated with a relatively lower MMR duration in months. These included prior treatment with Cabazitaxel (3.4 months), hepatic metastases (4.33 months), and peritoneal metastases (4.5 months) [Table 5].

**Figure 2:**
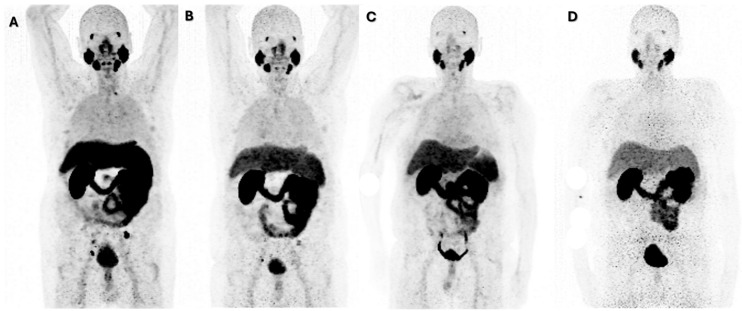
A 68-year-old male with Gleason score 7 prostate adenocarcinoma, BRCA germline negative, somatic negative. He had previous local treatment with brachytherapy and external beam radiation. The previous systemic therapy included enzalutamide and leuprorelin. A baseline [^68^Ga]Ga-PSMA PET/CT was performed in 2018, with PSA 0.70 ng/mL. The scan showed PSMA-avid bone and LNM and PSMA uptake in prostate bed (**A**), MIP. The patient was then treated with four cycles of [^177^Lu]Lu-PSMA PRLT. At three months post [^177^Lu]Lu-PSMA PRLT, a [^68^Ga]Ga-PSMA PET/CT was performed. The scan showed a good response in a majority of the previously seen PSMA-avid lesions in bone and soft tissue (**B**), MIP. A further two cycles of [^177^Lu]Lu-PSMA PRLT were administered for residual disease and a [^68^Ga]Ga-PSMA PET/CT was performed six months post these additional cycles of [^177^Lu]Lu-PSMA PRLT. PSA at time of scan was 0.10 ng/mL. The scan revealed a complete response (**C**), MIP. A follow-up [^68^Ga]Ga-PSMA PET/CT was performed at one year post six cycles of [^177^Lu]Lu-PSMA PRLT. The scan demonstrated persistent improvement with no PSMA-avid lesions and corresponding undetectable PSA (<0.02 ng/mL) (**D**), MIP. Hormonal therapy was subsequently discontinued. The patient remained in remission clinically, symptomatically, biochemically and on molecular imaging.

**Figure 3:**
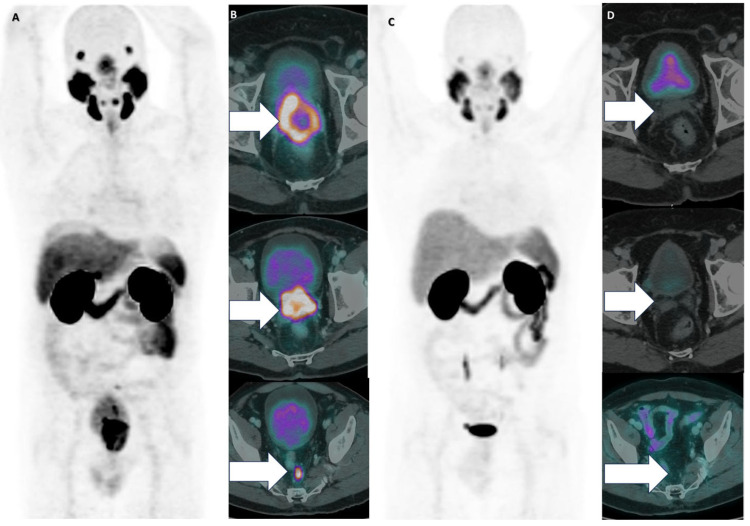
A 65-year-old male with Gleason score 10, plasmacytoid variant, MSI-H/PDL1 prostate adenocarcinoma. The patient was previously treated with goserelin and enzalutamide. A baseline [^68^Ga]Ga-PSMA PET/CT was performed with PSA 14.10 ng/mL (**A**), MIP. The axial slices with arrows show pelvic LNM and infiltrating prostate mass, locally advanced in large and small bowel and bladder (**B**), The patient was treated with four cycles of [^177^Lu]Lu-PSMA PRLT. [^68^Ga]Ga-PSMA PET/CT was performed at six months post four cycles of [^177^Lu]Lu-PSMA PRLT (**C**)**,** MIP. The axial slices with arrows highlight a remarkable complete response in all tumor sites (**D**). The patient had complete clinical, molecular and biochemical response with no new symptoms for 48 months.

**Figure 4:**
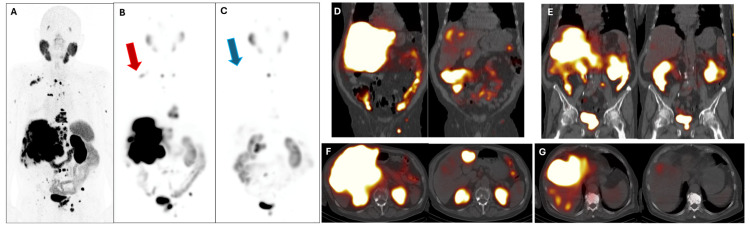
The 80-year-old patient was referred to PRLT due to mCRPC with neuroendocrine differentiation (Gleason score 9). The patient had received local therapy of the primary tumor after diagnosis (cT4, 2014), therapy with goserelin (2016–2020, resumed from 2022 due to local recurrence with rectal infiltration, bone, and LNM), enzalutamide, and denosumab. In August 2024, a rising PSA level (up to 71 ng/mL) prompted a [^68^Ga]Ga-PSMA PET/CT (**A**), MIP, which showed progression with local recurrence, extensive bilobar liver metastases, disseminated bone metastases, and LNM. Consequently, the patient was administered the first cycle of [^177^Lu]Lu-PSMA (9.0 GBq) combined with benfo-oxythiamine, which was well tolerated (beginning of August 2024; PSA 79.5 ng/mL). Post-therapeutic SPET/CT the day after injection (**B**), MIP revealed intense bilobar hepatic PSMA uptake («sink effect», reflecting intense PSMA accumulation in liver metastases) and uptake in bone metastases and LNM (e.g., red arrow indicating retroclavicular on the right). At the end of September 2024, the patient was administered the second cycle of [^177^Lu]Lu-PSMA PRLT (8.6 GBq, PSA 55.1 ng/mL); post-therapeutic SPET/CT (**C**), MIP demonstrated an overall extreme response with reduction of liver size (craniocaudal dimension decreased from 20.5 cm to 18.5 cm) and dramatic decrease of hepatic uptake of the radiotherapeutic compound. Most bone metastases and LNM showed a marked decrease in PSMA uptake (e.g., blue arrow indicating the absence of retroclavicular LNM), with only a few lesions visible at imaging (“molecular imaging response”). Coronal PET/CT (**D**,**E**), on the left, August 2024; on the right, September 2024, shows the extreme response of hepatic metastases and LNM. Axial PET/CT (**F**,**G**), on the left, August 2024; on the right, September 2024, confirmed the massive reduction of uptake by bilobar hepatic lesions.

**Figure 5:**
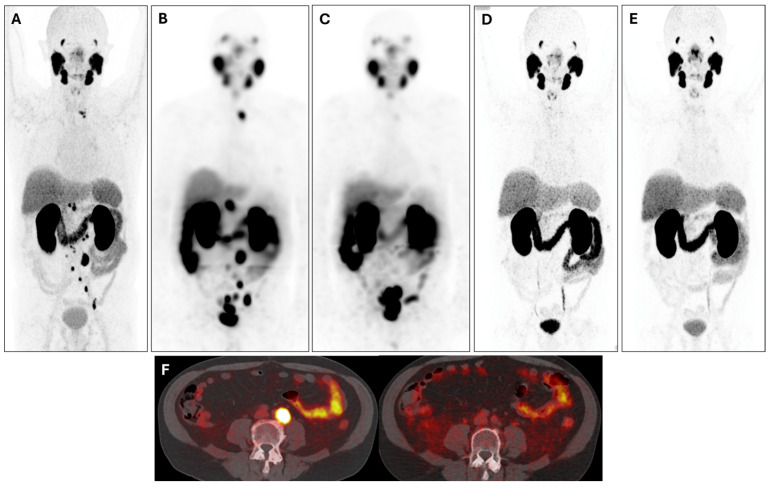
The 71-year-old patient was diagnosed with cribriform prostate adenocarcinoma (Gleason score 7b) in 2002 (pT2a pN0 M0). He was treated with prostatectomy and lymphadenectomy (2002), and due to biochemical (PSA up to 2 ng/mL) and local recurrence (MRI), therapy with bicalutamide (2010) and intensity-modulated radiotherapy (2011) were performed. During follow-up, [^68^Ga]Ga-PSMA PET/CT in September 2021 (**A**), MIP, showed multiple supra- and infra-diaphragmatic LNM; therefore, bicalutamide was stopped. The first cycle of PRLT with 9.0 GBq of [^177^Lu]Lu-PSMA was administered in December 2021 (pre-PRLT PSA: 67.4 ng/mL) and the second cycle with 7.5 GBq of [^177^Lu]Lu-PSMA in February 2022; both cycles were well tolerated. PSA showed 85.7% decrease after one cycle; biochemical response was confirmed by molecular imaging criteria, comparing the post-therapeutical SPET/CT after the first PRLT (**B**), MIP, and after the second PRLT (**C**), MIP. [^68^Ga]Ga-PSMA PET/CT performed in August 2022 (**D**), MIP, and in August 2023 (**E**), MIP, further confirmed complete remission of the disease. Axial PET/CT images (**F**), on the left, September 2021; on the right, August 2022, show the reduction of PSMA uptake by para-aortic LNM. The patient is still alive, with last PSA 1.44 ng/mL (June 2024, 97.8% of decrease compared to pre-PRLT values).

The WARMTH multicenter study [11] found that chemotherapy-naïve mCRPC patients receiving [^177^Lu]Lu-PSMA-617 therapy had a significantly longer OS than patients with a history of chemotherapy (taxane-based chemotherapy lines). This was related to cellular remodeling and the formation of treatment-resistant clones during chemotherapy [12]. Higher PSA levels have been reported to be associated with higher mortality [13], and lower PSA levels have been associated with greater disease progression in high-grade locally advanced prostate cancer [14]. There are factors that may influence the absolute effect of [^177^Lu]Lu-PSMA PRLT in patients. This has been reported with tumor metabolic heterogeneity in discordant PSMA and FDG PET/CT uptake and distribution [15]. Several studies suggest that the site of metastases may influence OS [11,15,16], with lung and liver metastases often linked to worse outcomes compared to bone and non-visceral metastases. However, there are studies that also show that extreme response to [^177^Lu]Lu-PSMA PRLT is not dependent on the metastatic site (bone, lymph nodes, or parenchymal tissues) [1,4,17,18]. Figure 2, Figure 3, Figure 4 and Figure 5 show some representative cases of our series.

Our analysis suggests that organized intrinsic mechanisms may drive extreme response. Tumor cells employ various mechanisms to evade immune detection and suppress antitumor responses [19,20]. However, there is growing evidence of immunomodulatory effects of ionizing radiation, resulting in an in situ “vaccination” effect [21,22,23,24]. The basic immune system comprises the innate (general) immune system and the adaptive (specialized) immune system [25]. The innate immune system includes natural killer (NK) cells, macrophages, dendritic cells, and neutrophils, as well as active molecules like the complement system in serum. The adaptive immune system includes lymphocytes and antibodies, which further recognize the structural details or amino acid sequences of foreign antigens with high precision. Both innate and adaptive immune systems, by operating in a coordinated manner, combat infectious diseases or cancer cells and molecules, allowing the tissue to return to its normal state [21,22,23,24]. Parker et al. [17] described in a case report two heavily pre-treated patients with mCRPC with an extraordinary response to [^177^Lu]Lu-PSMA PRLT, aged 73 and 80, showing progression of the disease with diffuse bone involvement and bone and soft tissue metastases, respectively. Both patients showed an exceptional long-term, complete response to [^177^Lu]Lu-PSMA PRLT. Zhang et al. [1] presented a case of mCRPC progressing after multiple lines of therapy and presenting with lung, lymph node, and extensive bone metastases. The patient underwent [^177^Lu]Lu-PSMA PRLT and had excellent response to treatment, especially with complete regression of lung metastases.

The “abscopal effect” [8] may play a crucial role in antitumor effect of PRLT; it suggests several immune-mediated mechanisms through which local radiation disrupts the tumor microenvironment, enhances tumor antigen presentation, and stimulates antitumor activity at distant tumor sites. If EBRT targets a limited number of tumor lesions, leading to a more localized antitumor effect, RLT delivers ionizing radiation directly to cancer cells through a carrier molecule (e.g., antibodies or small molecules) targeting specific tumor-associated antigens or receptors independently of their site. This precise targeting allows for the destruction of tumor cells across multiple lesions with minimal damage to healthy tissue [26,27]. RLT has been shown to enhance the “abscopal effect”, with increasing evidence of robust adaptive immune responses and development of immunological memory. Ionizing radiation, whether from α or β-emitting nuclides, has immunomodulatory effects, boosting antitumor immunity [28,29,30,31,32,33,34,35,36]. This process enhances both the presentation of tumor antigens and the activation of the immune system, leading to immunogenic cell death (ICD) and more effective, sustained tumor destruction [9,37,38,39,40]. Immune system activation appears to be a primary factor in enhanced responses. Other potential mechanisms, such as metabolic and genetic factors, which may contribute and influence these outcomes, would warrant further investigation to fully understand their impact.

ICD is one of several mechanisms by which tumor cell death is induced through antitumor therapy. This process involves changes in cell surface composition and a regulated release of soluble mediators. The key agents driving these signaling processes are receptors expressed by dendritic cells, which facilitate the presentation of tumor antigens to T cells. Tumor cells undergoing ICD can further stimulate an adaptive anti-cancer immune response that targets any remaining cancer cells sharing the same antigenic profile. A major pathway proposed for ionizing radiation-induced ICD is linked to DNA damage, as the cytotoxic effects are largely mediated by unrepaired DNA double-strand breaks [41,42,43,44,45]. These DNA damage fragments are recognized as endogenous damage-associated molecular patterns (DAMPs), which are transported to the cytosol. There, they trigger the release of tumor-associated antigens (TAAs), proinflammatory cytokines (including type I interferons, IFN-γ, TNF-α), uric acid, heat shock proteins, high-mobility group box 1 (HMGB1), calreticulin, annexin-A1, and chemokines (e.g., CXCL16 and MCP-1). A key mediator in this process is the DNA-sensing enzyme cyclic GMP-AMP synthase (cGAS), which detects cytosolic DNA and activates the stimulator of interferon genes (STING)-dependent pathway. The resulting type I IFN response is crucial for dendritic cell function and plays a central role in activating the adaptive immune response [31,41,42,43,44,45].

It has been shown that radiation can enhance antitumor responses by promoting the activation and antigen-presenting capabilities of dendritic cells. Ionizing radiation induces DNA damage in tumor cells at the targeted site, triggering the release of TAAs, proinflammatory cytokines, and chemokines as previously described. Radiation also induces the release of danger signals, such as HMGB1, ATP, and translocation of calreticulin to the tumor cell surface. This process upregulates cell adhesion molecules (ICAM-1 and VCAM-1), the death receptor Fas, and MHC I and II, thereby enhancing the recruitment and activation of innate immune cells and tumor-specific lymphocytes [42,43,44,45]. The release of these mediators activates circulating dendritic cells, which take up dying tumor cells, migrate to lymph nodes, and present tumor antigens to naïve CD4+ T cells. With appropriate costimulatory signals (e.g., IFN-γ and IL-12), dendritic cells promote the activation and expansion of effector CD4+ T cells. These T cells assist in activating CD8+ cytotoxic T lymphocytes, which play a central role in tumor cell apoptosis through Fas-mediated and granzyme/perforin mechanisms. Additionally, dendritic cells can cross-present tumor antigens to cytotoxic T lymphocytes, further enhancing the antitumor immune response. This cascade effectively engages inflammatory and immune cells, triggering a robust and long-lasting response [21,22,23,24,43,44,45]. Moreover, benfo-oxythiamine (BOT) might even enhance these effects [46].

## 5. Conclusions

In our dual-center case series, we found that extreme response in mCRPC patients treated with [¹⁷⁷Lu]Lu-PSMA PRLT was independent of tumor site, pattern of disease, radiotherapeutical uptake, baseline PSA levels, previous treatments, age, race/nationality, Gleason score, and BRCA sequencing status. There was no direct association observed between the [¹⁷⁷Lu]Lu-PSMA dose or number of therapy cycles to patient extreme response. Patients demonstrated an exceptional, enhanced response following [¹⁷⁷Lu]Lu-PSMA PRLT, with notable improvements biochemically, clinically, and on imaging, with a prolonged therapeutic effect (longest duration of maintained response of 99 months and a mean of 17.44). After analyzing various clinical factors within our dual-center cohort and reviewing existing research, we hypothesize that this extreme response effect may be attributed to the immunomodulatory enhancement of ionizing radiation, which facilitates antigen presentation, activates the immune system, and promotes tumor cell destruction. This may be one of the primary contributing factors to the prevention of cellular remodeling, avoiding the clustering of treatment-resistant clones and highlighting the significant role of PRLT in the early management of mCRPC. However, the potential influence of metabolic and genetic variables as contributing factors should not be overlooked. These variables, along with the immunomodulatory effects of ionizing radiation, warrant further quantitative investigation through larger cohort studies, with particular emphasis on genes related to DNA repair.

## Figures and Tables

**Table 1:** Baseline clinical variables of patients treated at time of presentation of PRLT.

		Age at the Time of Presentation for PRLT	No. of Cycles to Response	No. of Months of Maintained Response	Baseline PSA (ng/mL)	PSA (ng/mL) at Response
Mean		71.78	2.81	17.44	175.15	31.51
Std. Deviation		8.529	1.238	21.836	329.263	72.958
Percentiles	25	65.25	2.00	4.00	9.44	0.33
	50	71.00	3.00	11.00	56.95	2.51
	75	79.50	3.00	17.00	139.75	16.63

**Table 2:** Paired *t*-test PSA baseline vs. PSA at response.

	Paired Differences					T	df	Significance	
	Mean	Std. Deviation	Std. Error Mean	95% Confidence Interval of the Difference				One-Sided *p*	Two-Sided *p*
				Lower	Upper				
Baseline PSA (ng/mL)—PSA (ng/mL) at response	143.64	274.920	45.820	50.627	236.666	3.135	35	0.002	0.003

**Table 3:** Association of clinical variables with number of months of maintained response (n = 36).

Variable	Number of Months of Maintained Response			*p*-Value
	<6 Months	6–<12 Months	≥12 Months	
Age at presentation	70.91 ± 9.62	75.70 ± 7.97	69.80 ± 7.68	0.224
Gleason score	4.64 ± 3.0	4.20 ± 2.89	4.13 ± 1.76	0.871
Number of cycles to response	2.82 ± 1.53	3.0 ± 0.94	2.67 ± 1.23	0.813
Baseline PSA	390.74 ± 506.4	113.46 ± 186.9	58.18 ± 106.9	0.026
PSA at response	88.91 ± 112.3	13.20 ± 27.70	1.62 ± 2.75	0.004

**Table 4:** Number of [^177^Lu]Lu-PSMA PRLT cycles and frequency to response.

No. of Cycles (n)	All Patients (n = 36)	Frequency %
1	4	11.1%
2	12	33.3%
3	12	33.3%
4	5	13.9%
5	1	2.8%
6	2	5.6%

**Table 5:** Baseline clinical variables of patients: prior therapies and pattern of disease. (*) Mean PSA value was estimated excluding single patient with aberrant PSA increase post-[^177^Lu]Lu-PSMA PRLT.

Previous Treatment for mCRPC	All Patients n = 36	Mean Maintained Response (Months)	Max. Maintained Response (Months)	Mean Decline in PSA (%)	*p*-Value
Docetaxel	7 (19.4%)	17	83	78.14	0.172
Cabazitaxel	5 (13.9%)	3.4	5	74.40	
Abiraterone	10 (28%)	17.2	83	85.60	
Enzalutamide	11 (31%)	6.45	14	81.18	
EBRT	15 (41.7%)	11.45	48	84.14 (*)	
Radium-223 dichloride	1 (2.7%)	14	14	99.0	
Pattern of disease:					
Bone metastases	27 (75%)	15.1	99	85.05	0.721
Lymph node metastases	27 (75%)	17.15	99	84.48 (*)	
Liver metastases	6 (16.67%)	4.33	11	66.0	
Peritoneal metastases	2 (5.6%)	4.5	5	69.50	

**Table 6:** Association of Gleason score vs. number of months of maintained response.

Gleason Score (GS)		Maintained Response (Months)			
GS	Frequency	Mean	Std. Deviation	Minimum	Maximum
7	11	25.64	22.85	6	83
8	8	9.13	6.03	2	19
9	6	8	6.03	2	17
10	5	33.2	41.07	4	99
unknown	4	5.25	3.59	2	10
6	2	19	24.04	2	36

**Table 7:** Patient subgroup with known BReast CAncer gene (BRCA) sequencing status and association of maintained response to [^177^Lu]Lu-PSMA PRLT and %PSA decline post-[^177^Lu]Lu-PSMA PRLT response.

Known BRCA Status	Patients (n = 12)	Mean Maintained Response (Months)	Max. Maintained Response (Months)	Mean Decline in PSA (%)
BRCA1/2 wild-type	6 (50%)	6.67	13	77.0
BRCA 1/2 negative	1 (8.33%)	7	7	94.0
BRCA germline negative and somatic positive	1 (8.33%)	36	36	50.0
BRCA germline negative, Ssomatic negative	2 (16.67%)	27	48	74.5
BRCA 2 positive	2 (16.67%)	43	83	92.18

**Table 8:** Patient subgroup data with ≥12 months progression-free/maintained therapeutic response compared with prior therapies, Gleason score, pathology and sequencing, pattern of disease, number of cycles to response, maintained response (months), and %PSA decline.

Prior Therapies	Gleason Score; Specific Pathology and Sequencing	Pattern of Disease	No. of Cycles to Response	Maintained Response (Months)	%PSA Decline
Bicalutamide, Enantone	9 BRCA1/2 wild-type	Bone, LNM, Adrenal	1	12	99
Goserelin, Abiraterone, Enzalutamide, Zoledronic Acid, Cabazitaxel, EBRT (Thoracic Spine, Prostate + Left Ilium)	8	Bone, LNM	4	12	89
Bicalutamide, Prostatectomy + EBRT, Lymphadenectomy, Orchiectomy, Abiraterone, Enzalutamide + Trenantone	8 BRCA 1/2 wild-type	Bone, LNM, Adrenal, Gerota Fascia	1	13	77
Prostatectomy + EBRT, Trenantone + Abiraterone, EBRT Ilium, Enzalutamide	8	LNM, Bone, Pleura	2	13	98
Irreversible Electroporation (IRE)	7	Left seminal vesicle, LNM	2	14	85
Prostatectomy + Lymphadenectomy, EBRT (Prostate, Rib + T4), Radium-223 dichloride, Trenantone, Enzalutamide, Bicalutamide, Denosumab	7	Bone	3	14	99
Bicalutamide, Prostate Electroporation with Bleomycin	7	Local recurrence, LNM,	2	15	100
Da Vinci Prostatectomy, Bicalutamide, Abiraterone	9 Dedifferentiated Adenocarcinoma VUS CHEK2	LNM, Bone	2	17	97
IRE	7	LNM	3	17	89
Prostatectomy, Salvage EBRT of Prostate Bed and Pelvic Lymph Pathways, Leuprorelin, Apalutamid, Darolutamid	8	LNM	2	19	−683
Prostatovesiculectomy + LA, Bicalutamide, IMRT prostate bed	7 Prostatic cribriform adenocarcinoma	LNM	2	32	86
Brachytherapy, Bicalutamide, LHRH Analogue, Interstitial HDR-Afterloading Brachytherapy + Boost	7	Local recurrence + seminal vesicles + dorsal bladder wall, LNM	2	33	72
Prostatectomy, Goserelin	6 Invasive prostate acinar adenocarcinoma BRCA germline negative, somatic positive, MSS/PDl1	Prostate bed, locally advanced	2	36	50
Brachytherapy, EBRT, Enzalutamide, Leuprorelin	7 BRCA germline negative, somatic negative	Bone, LNM, prostate	6	48	86
Goserelin, Enzalutamide	10Plasmacytoid variant MSI-H/PDL1	LNM, Prostate, Locally advanced in bowel and bladder	4	48	100
Degarelix, Denosumab, 6x Docetaxel, Leuprorelin, Abiraterone	7BRCA 2, TMB 10.53 mut/Mb, VUS CHEK2, CDH1	Bone and bone marrow	4	83	96
Bicalutamide, Da Vinci Prostatovesiculectomy + Lymphadenectomy, Buserelin, Leuprorelin, Finasterid	10 Acinar partial neuroendocrine differentiation, immunostaining positive for synaptophysin, CgA, CD-56	Local recurrence, LNM, Bone	3	99	100

## Data Availability

The raw data supporting the conclusions of this article will be made available by the authors upon request.

## Supplementary Materials

The following supporting information can be downloaded at: https://www.mdpi.com/article/10.3390/cancers17030476/s1, Table S1: Patient summary (n = 36) data sheet with associated clinical variables.
