# Supplementary material for: Is Extraordinary Response and Long-Term Remission of Metastatic Castration-Resistant Prostate Cancer (mCRPC) After [¹⁷⁷Lu]Lu-PSMA Radioligand Therapy Due to an Immunomodulatory Effect (Radiovaccination)? A Dual Center Experience on Super-Responders
Source: Cancers (Basel). 2025 Jan 31;17(3):476. doi: 10.3390/cancers17030476 (PMC11815733; doi:10.3390/cancers17030476)
Supplement: Supplementary file 1 [file cancers-17-00476-s001.zip › cancers-3388310-supplementary.pdf]

Table S1: Patient summary (n=36) data sheet with associated clinical variables. Age is at time of presentation for PRLT. PSA @ baseline of PRLT; PSA @response to PRLT. LNM[1] lymph node metastases; EBRT[2] External Beam Radiation Therapy; BRCA[3] Breast Cancer Gene; IMRT[4] Intensity-modulated radiation therapy; IRE[5] Irreversible Electroporation.

Age	Prior Therapies	Gleason score; Specific pathology and sequencing	Pattern of disease	No. of cycles to response	Maintained response (months)	Baseline PSA (ng/mL)	PSA (ng/mL) @ response
**66**	6x Docetaxel, Trenantone, Denosumab, Enzalutamid, Abirateron, 3x Docetaxel, Cabazitaxel,	10, Prostatic adenocarcinoma, BRCA 1/2 wild type	Seminal vesicles, LNM, bone and bone marrow	5	4	145	67.3
**55**	Bicalutamid + Trenantone, Zometa	8 (4+4), Prostatic adenocarcinoma, BRCA 2	LNM, bone	3	3	1219	142
**62**	Bicalutamid, DaVinci Prostato-vesiculectomy + Lymphadenectomy, Buserelin, Leuprorelin, Finasterid	10 (5+5), Acinar partial neuroendocrine differentiation, Immunostaining positive for Synaptophysin, CgA, CD-56	Local recurrence, LNM, bone	3	99	356.2	0
**63**	Bicalutamid, Enantone	9 (4+5), Prostatic adenocarcinoma BRCA1/2 wild type	Bone, LNM, adrenal gland	1	12	7.6	0.76
**58**	Bicalutamid, Prostate electroporation with Bleomicin	7b (4+3), Prostatic adenocarcinoma	Local recurrence, LNM,	2	15	92	0.05
**75**	Bicalutamid, Prostatectomy + EBRT, Lymphadenectomy, Ochectomy, Abiraterone, Enza + Trenantone	8 (4+4), Prostatic adenocarcinoma, BRCA wild type	Bone, LNM, adrenal gland, Gerota Fascia	1	13	5.48	1.24
**75**	Bicalutamide, Leuprorelin	9, Prostatic adenocarcinoma, no evidence of genetic tumor syndrome (NGS)	LNM, bone	3	10	196	3.25
**78**	Brachytherapy, Bicalutamid, LhRH Analogue, Interstitial HDR-Afterloading Brachytherpy + Boost	7b (4+3), Prostatic adenocarcinoma	Local recurrence + Seminal vesicles + dorsal bladder wall, LNM	2	33	2.68	0.76
**68**	Brachytherapy, EBRT, Xtandi, Lucrin	7,	Bone, LNM, prostate	6	48	0.70	0.10

		Prostatic adenocarcinoma, BRCA Germline negative, Somatic negative					
**81**	Brachytherapy, Zoladex, Xtandi	7, Prostatic adenocarcinoma, BRCA Germline negative, Somatic negative	Prostate bed, LNM	4	6	10.42	3.99
**86**	Buserelin, Enzalutamid, EBRT (prostate bed + LNM)	8, Prostatic adenocarcinoma	Bone	2	4	46.6	0.86
**71**	DaVinci Prostatectomy, Bicalutamid, Abiraterone	9 (4+5), Dedifferentiated prostatic adenocarcinoma, VUS CHEK2	LNM, bone	2	17	0.209	<0.006
**76**	Degarelix/ Firmagon, Denosumab, 6x Docetaxel, Lupron, Abiraterone	7b (4+3), Prostatic adenocarcinoma, BRCA 2, TMB 10.53 mut/Mb, VUS CHEK2, CDH1	Bone and bone marrow	4	83	9,42	0.37
**77**	Finasterid, Autologous DC-Vaccination, loco-regional Radiofrequency-Hyperthermia (mLHT), trans-urethreal Hyperthermy with oncolytic Virotherapy (NDV), loco-regional Electro-Hyperthermia (eEHT), Irreversible Electroporation (IRE[5]) with Bleomycin	9 (4+5), Prostatic adenocarcinoma	LNM, bone	1	2	87.1	18.8
**65**	Gold seeds implantation, Bicalutamid, degarelix, abiraterone, EBRT (Prostate, ribs, corpus cavernosum; radiosurgery of brain mets)	8 (4+4), Prostatic adenocarcinoma, BRCA1/2 negative	LNM, brain, lung, bone, corpus cavernosum	3	7	67.3	4.1
**81**	Goserelin, Abiraterone, Enzalutamid, Zoledronic Acid, Cabazitaxel, EBRT (thoracic spine, prostate + left os ilium)	8, G3, Prostatic adenocarcinoma	Bone, LNM	4	12	90	11.1
**69**	IMRT[4] prostate, 6x Docetaxel, Enza + Trenantone, 6x Capacitaxel	10, Prostatic adenocarcinoma, BRCA wild type	LNM, bone, hepatic, soft tissue, peritoneal	3	4	99.9	2.8
**69**	ADT (unknown); IRE	7b (4+3), Prostatic adenocarcinoma	Local invasion left seminal vesicle, LNM	2	14	15	2.23

**68**	ADT (unknown); Irreversible Electroporation (IRE)	7a (3+4), Prostatic adenocarcinoma	LNM	3	17	37	3.92
**78**	ADT (unknown); Laminectomy and EBRT (L2, L3, L4)	Gleason unknown, Prostatic adenocarcinoma	Prostate, LNM, bone	6	3	124	15.1
**72**	Leuprorelin, zoledronic acid, 6x Docetaxel, enzalutamide, 6x Cabazitaxel	Gleason unknown, Prostatic adenocarcinoma, BRCA 1/2 wild type	Bone and bone marrow	3	2	29	2.0
**87**	Prostate brachytherapy and EBRT, Leuprorelin, abiraterone, 6x Docetaxel, EBRT (lumbar spine)	Gleason unknown, Prostatic adenocarcinoma	Bone	3	10	619	90.5
**71**	Prostate electroporation with Bleomicin, denosumab	7b (4+3), Prostatic adenocarcinoma	Local recurrence, LNM, bone	3	11	2.6	0.313
**82**	Prostate electroporation with Bleomicin, trenantone, enzalutamide, denosumab, EBRT (C5-C7)	G 7b (4+3), Prostatic adenocarcinoma	Bone	2	9	17	0.61
**80**	Prostate HIFU, Goserelin, Enzalutamid, Denosumab	9, Prostatic adenocarcinoma with neuroendocrine differentiation	Bilobar hepatic, bone and LNM; prostate	1	2	79.5	55.1
**82**	Prostatectomy + EBRT, Trenantone + Abiraterone, EBRT Ilium, Xtandi	8 (4+4), Prostatic adenocarcinoma	LNM, bone, pleura	2	13	263	5.4
**71**	Prostatectomy + LA, Degarelix, bicalutamide, abiraterone, Zoledronic acid, Cisplatin/Etoposid, Docetaxel, EBRT (Sacrum)	10, Prostatic adenocarcinoma with neuroendocrine differentiation, CgA and Synaptophysin positive staining	Bone, LNM, hepatic, adrenal	4	11	84	17.14
**74**	Prostatectomy + Lymphadenectomy, Leuprorelin, abiraterone, 4x Cabazitaxel, EBRT (Prostate bed + LN, hip peri-prosthetic)	8, Prostatic adenocarcinoma	Bone, hepatic	2	2	1345	186
**67**	Prostatectomy + Lymphadenectomy, lung hyperthermia + wedge resection + hilar/interlobular lymphadenectomy, intratumoral immune stimulation via Viscum injection, Bicalutamide, leuprorelin, enzalutamide, Zoledronic acid,	6 (3a+3b), moderately differentiated tubular and small glandular prostatic adenocarcinoma	Bone, LNM, adrenal and lung, hepatic	3	2	919	369

	EBRT (prostate bed), whole-body + right shoulder hyperthermia						
**82**	Prostatectomy + Lymphadenectomy, EBRT (Prostate, Rib + T4), Xofigo, Trenantone, Enzalutamid, Bicalutamid, Denosumab	7a (3+4), Prostatic adenocarcinoma, no clinically relevant mutations	Bone	3	14	9.48	0.06
**56**	Prostatectomy with lymphadenectomy (LA), EBRT (prostate bed), cyberknife of right external iliac LNM, 18x Docetaxel, 8x Cabazitaxel, Eligard	9 (4+5), Prostatic adenocarcinoma with cribriform aspects, BRCA1/2 wild type	Hepatic, bone, peritoneal carcinosis	2	5	204	119
**59**	Prostatectomy, Salvage EBRT of Prostate bed and pelvic Lymph pathway, Eligard, Apalutamid, Darolutamid	8 (4+4), Prostatic adenocarcinoma	LNM	2	19	<0.06	0.41
**63**	Prostatectomy, Zoladex	6, Invasive prostatic acinar adenocarcinoma, BRCA Germline negative, Somatic positive, Mss/PDl1	Prostate bed, locally advanced	2	36	0.04	<0.02
**71**	Prostatovesiculectomy + Lymphadenectomy, Bicalutamid, IMRT Prostate bed	7b (4+3), Prostatic cribriform adenocarcinoma	LNM	2	32	67.4	9.65
**81**	Transurethral prostate hyperthermy + Enantone, Cyberknife Radiotherapy, Enzalutamid + Denosumab	Gleason unknown, Prostatic adenocarcinoma	LNM, bone	3	6	40.7	0.275
**65**	Zoladex, Xtandi	10, Plasmacytoid variant prostatic adenocarcinoma, MSI-H/PDL1	LNM, prostate, locally advanced in bowel and bladder	4	48	14.10	<0.01
